# Cationized liposomal keto-mycolic acids isolated from *Mycobacterium bovis bacillus* Calmette-Guérin induce antitumor immunity in a syngeneic murine bladder cancer model

**DOI:** 10.1371/journal.pone.0209196

**Published:** 2019-01-04

**Authors:** Takayuki Yoshino, Jun Miyazaki, Takahiro Kojima, Shuya Kandori, Masanobu Shiga, Takashi Kawahara, Tomokazu Kimura, Takashi Naka, Hideyasu Kiyohara, Miyuki Watanabe, Sho Yamasaki, Hideyuki Akaza, Ikuya Yano, Hiroyuki Nishiyama

**Affiliations:** 1 Department of Urology, Faculty of Medicine, University of Tsukuba, Ibaraki, Japan; 2 Department of Urology, International University of Health and Welfare, Chiba, Japan; 3 Department of Food and Nutrition, Faculty of Contemporary Human Life Science, Tezukayama University, Nara, Japan; 4 Japan BCG Laboratory, Kiyose, Japan; 5 Department of Molecular Immunology, Research Institute for Microbial Diseases, Osaka University, Osaka, Japan; 6 Division of Molecular Immunology, Medical Institute of Bioregulation, Kyushu University, Fukuoka, Japan; 7 Department of Molecular Immunology, Immunology Frontier Research Center, Osaka University, Osaka, Japan; 8 Division of Molecular Immunology, Medical Mycology Research Center, Chiba University, Chiba, Japan; 9 Strategic Investigation on Comprehensive Cancer Network, University of Tokyo, Tokyo, Japan; 10 Osaka City University, Osaka, Japan; Laurentian, CANADA

## Abstract

Intravesical therapy using *Mycobacterium bovis bacillus* Calmette-Guérin (BCG) is the most established cancer immunotherapy for bladder cancer. However, its underlying mechanisms are unknown. Mycolic acid (MA), the most abundant lipid of the BCG cell wall, is suspected to be one of the essential active components of this immunogenicity. Here, we developed cationic liposomes incorporating three subclasses (α, keto, and methoxy) of MA purified separately from BCG, using the dendron-bearing lipid D22. The cationic liposomes using D22 were efficiently taken up by the murine bladder cancer cell line MB49 in vitro, but the non-cationic liposomes were not. Lip-kMA, a cationic liposome containing keto-MA, presented strong antitumor activity in two murine syngeneic graft models using the murine bladder cancer cell lines MB49 and MBT-2 in comparison to both Lip-aMA and Lip-mMA, which contained α-MA and methoxy-MA, respectively. Interestingly, Lip-kMA(D12), which was made of D12 instead of D22, did not exhibit antitumor activity in the murine syngeneic graft model using MB49 cells, although it was successfully taken up by MB49 cells in vitro. Histologically, compared to the number of infiltrating CD4 lymphocytes, the number of CD8 lymphocytes was higher in the tumors treated with Lip-kMA. Antitumor effects of Lip-kMA were not observed in nude mice, whereas weak but significant effects were observed in beige mice with natural killer activity deficiency. Thus, a cationized liposome containing keto-MA derived from BCG induced in vivo antitumor immunity. These findings will provide new insights into lipid immunogenicity and the underlying mechanisms of BCG immunotherapy.

## Introduction

Worldwide, urothelial bladder cancer is the 7th most common cancer in men and the 17th most common in women [1]. Approximately 75% of the patients with this cancer present with non-muscle invasive bladder cancer (NMIBC) at the time of diagnosis [1]. NMIBCs are treated by transurethral resection of the bladder tumor (TURBT), but the relatively high rates of recurrence and progression to muscle-invasive disease are major concerns in the treatment of NMIBC [2]. *Mycobacterium bovis bacillus* Calmette-Guérin (BCG) is a live vaccine for tuberculosis. In 1976, Morales et al. reported that a topical application of high-dose BCG to the bladder lumen exerted powerful preventive effects against bladder cancer recurrence [3]. Since then, intravesical BCG therapy has been widely used and is recognized as the standard treatment to prevent the recurrence and progression of NMIBC [2].

Despite its efficacy, intravesical BCG therapy is associated with various adverse events such as urinary frequency, fever, and granulomatous prostatitis [4, 5]. In addition, because BCG is an attenuated live bacterium, BCG therapy can result in an uncommon but possibly fatal systemic infection and an immune reaction. BCG sepsis is the most serious adverse event. More than ten deaths due to BCG sepsis have been reported since 2006 [4]. To avoid such unfavorable adverse events, it is necessary to develop a more active but less toxic immunotherapeutic agent.

To improve BCG therapy, the underlying mechanisms must be clarified. Although the precise mechanisms are not yet established, the attachment and internalization of BCG into bladder cancer cells and the urothelium are speculated to take part in the initiation of multiple mechanisms involved in BCG-induced antitumor immunity [6, 7]. The attachment of BCG to bladder cancer or the urothelium is considered to be the first step in the induction of antitumor effects [6, 7]. The glycosaminoglycan layer, which covers the urothelium and is highly negatively charged, protects the urothelium from BCG and other bacteria [8, 9]. After its attachment and internalization into bladder cancer cells, BCG—and especially the BCG cell wall components—can induce the secretion of interleukin (IL)-6 and various other cytokines from bladder cancer cells, and the cytokines induce antitumor immunity [10–12].

BCG cell wall components such as the BCG-cell wall skeleton (CWS) and trehalose 6,6'-dimycolate have long been investigated as important immunogenic factors. The BCG cell wall components consist of highly characteristic hydrophobic molecules, including mycoloyl glycolipids, mannose-containing lipoglycans, and the cell wall skeleton. Indeed, the BCG cell wall components have been shown to stimulate Th-1-type immune responses through the production of several cytokines in animal models [13–16]. However, the clinical use of BCG cell wall components is limited because of difficulties relating to solubility and stability. Moreover, the negative surface charge causes poor cellular association [17].

Among the cell wall components, mycolic acids (MAs) are the most characteristic component of acid-fast bacteria such as *Mycobacterium tuberculosis* and the related species [18]. Although the structures of MAs vary greatly among mycobacterial species, they are basically very high-molecular-weight fatty acids with a long alkyl branched chain at the 2-position and a hydroxyl group at the 3-position, and MAs play a pivotal role in the highly hydrophobic barrier function of acid-fast bacterial cell walls [19, 20]. Several studies have demonstrated that MA is presented on CD1 molecules and is recognized by CD1-restricted T cells [21–23]. MA-liposomes have been shown to elicit inflammation in the mouse respiratory system [24]. However, due to the amphipathic properties of MAs and the large size and high complexity of the MA molecules, the pharmacological development of MAs has not succeeded, and there have been no reports on the antitumor activity of these agents.

Generally, mycobacteria contain several subclasses of MAs, which differ with respect to the polar functional group in the meromycoloyl chain. *M*. *tuberculosis* and BCG have three subclasses of MAs with different structures: α-MA, methoxy-MA, and keto-MA. Interestingly, the host immune response against the MAs of *M*. *tuberculosis* differs among subclasses [25]. In the present study, we hypothesized that the antitumor immune response would be elicited if we could successfully deliver MAs into cancer cells or immune cells by using an appropriate vector. We developed cationic liposomes incorporating the three subclasses of MA purified separately from BCG with the use of the dendron-bearing lipid D22. Our experimental results demonstrated that liposome cationized using keto-MA induces a distinctive tumor cell growth retardation in vivo through T-cell-dependent antitumor immunity. To our knowledge, this is the first report to demonstrate that purified and particular MA subclasses induce antitumor immunity in vivo.

## Materials and methods

### Materials and cells

*Mycobacterium bovis BCG* Tokyo 172 was kindly provided by Japan BCG Laboratory (Kiyose, Japan). N-hexane was purchased from Wako (Osaka, Japan). 1,2-Dioleoyl-sn-glycero-3-phosphocholine (DOPC) and cholesterol were purchased from Avanti Polar Lipids (Alabaster, AL). Dendron-bearing lipids D22 and D12 were purchased from Hygieia Bioscience (Osaka, Japan). Anti-mouse CD4 rat monoclonal antibody and anti-mouse CD8 monoclonal antibody were purchased from eBioscience (San Diego, CA).

Three transitional cell carcinoma cell lines were used: MBT-2 and MB49 were of murine origin, and T-24 was of human origin. MB49 was kindly donated by Dr. WT Godbey (Department of Chemical & Biomolecular Engineering, Tulane University), and MBT-2 and T-24 were from the National Institutes of Biomedical Innovation, Health and Nutrition in Japan.

The murine bladder cancer cell line MBT-2 and the human bladder cancer cell line T24 were maintained at 37°C in 5% CO_2_ in RPMI 1640 medium supplemented with 10% fetal bovine serum (FBS), penicillin, and streptomycin. The murine bladder cancer cell line MB49 was maintained in Dulbecco's Modified Eagle Medium (DMEM) supplemented as described for the MBT-2 and T24 cells.

### Animals

Female C57BL/6, C3H/HeN, and nude mice (Balb/c nu/nu) (6–7 weeks old) were purchased from Charles River Laboratories Japan (Yokohama, Japan). Beige mice (C57BL/6J-bg/bg) were purchased from RIKEN BRC (Tsukuba, Japan). The animals were kept in standard laboratory cages in groups of five per cage under a 12 h light/dark cycle in a temperature- and humidity-controlled environment with food and water ad libitum. Clinical symptoms including body weight, tumor bleeding, behavior, appearance and general health condition were monitored daily. All care and experimental procedures were performed in accordance with national and regional legislation on animal protection, and all animal procedures were consistent with the University of Tsukuba's Regulation of Animal Experiments and were approved by the Animal Experiment Committee, University of Tsukuba (reference numbers 17–434). For tissue histology, mice were sacrificed by cervical dislocation under isoflurane anesthesia (2%, 2 L/min).

### Isolation and purification of MA from heat-killed BCG cells by solvent fractionation

*Mycobacterium bovis* BCG Tokyo 172 was grown as a surface culture at 37°C in Sauton's medium for 9 days. The bacterial culture was harvested by centrifugation after autoclaving at 121°C for 15 min. Heat-killed packed cells (20 g) of *Mycobacterium bovis* BCG Tokyo 172 were fully hydrolyzed with 100 ml of 5N sodium hydroxide for 20 min at 121°C. After acidification to pH 2.0 with 5N hydrochloric acid, five volumes of n-hexane was added. The hexane layer was collected and evaporated. The n-hexane soluble fatty acids were washed twice with distilled water, and then the water-soluble layer was discarded, and the residual n-hexane soluble total fatty acids were weighed.

For the separation of crude MA, a small volume of chloroform (1 ml) was added to dissolve the total fatty acids, and then a large volume of methanol (50 ml) was added to precipitate the MA at 10°C overnight. For further purification, methanol precipitation was repeated once again, and the resultant methanol precipitate contained further purified MA.

### Thin-layer chromatographic preparation and the separation of MA subclasses

For the thin-layer chromatographic separation of MA, crude MA was methylated with 10% tri-methylsilildiazomethane in n-hexane for 30 min at room temperature. The methylester derivatives of total MAs were detected by thin-layer chromatography (TLC) on Silicagel G (Uniplate; 20x20 cm: Miles Scientific, Newark, DE), which was developed with n-hexane-diethyl ether (80:20, v/v; four runs). After the MA methylester subclass was located with iodine vapor, it was recovered from thin-layer plates with chloroform-methanol (9:1, v/v) [25]. Purified MA methylester was autoclaved with 3 ml of toluene, 5 ml of 5N sodium hydroxide, and 2 ml of ethanol. After acidification to pH 2.0 with 5N hydrochloric acid, n-hexane was added. The hexane layer was collected and evaporated to obtain purified MA.

### Mass spectrometric analysis of MA subclasses

The molecular weight of each MA methylester was determined by matrix-assisted laser desorption/ionization-time of flight (MALDI-TOF) mass spectrometry on a Voyager DE-STR workstation (Applied Biosystems, Foster City, CA) using 2.4-dihydroxybenzoic acid (2.5-DHB) as a matrix, as reported previously [26]. The samples were analyzed in the reflection mode with an accelerating voltage operating in the negative ion mode of 25kV. Next, 2.5-DHB at the concentration of 10 mg/ml was mixed with 1.0 ml of the matrix solution. The sample mixture was applied to the sample plate as a 1.0-ml droplet. The samples were then allowed to crystallize at room temperature. The MALDI-TOF mass spectra in the positive mode were acquired as described by Fujita et al. [26].

### The preparation of liposomes containing MA subclasses

We prepared three different liposomes, Lip-aMA, Lip-mMA, and Lip-kMA, each of which contains a different subclass of MA, i.e., α-, methoxy-, and keto-MA, respectively. The liposomes were prepared by dissolving 200 μg of α-, methoxy-, or keto-MA with DOPC, cholesterol, and the cationic dendron-bearing lipid D22 at the ratio presented in Table 1. The lipid mixture was dissolved in chloroform by mild heating at 60°C, and then the lipid thin-film was prepared by evaporating the solvents under nitrogen gas.

**Table 1:** Material ratio of liposomes with or without MA.

	MA (mg)	DOPC (mg)	Cholesterol (mg)	Dendrimer (mg)
Liposome with MA	0.2	1.8	0.2	0.066*
Lip-con	−	1.8	0.2	0.06*

The derived lipid film was recovered in 1 ml of phosphate-buffered saline (PBS) and sonicated for 15 min using a Biorupter (Cosmo Bio, Tokyo). The resultant liposome suspension was centrifuged at 14000 rpm for 30 min. The liposome pellet was then washed with PBS and applied to 10 freeze- and thaw- cycles in liquid nitrogen, followed by mini-extrusion (11 times) through a polycarbonate membrane with a pore size of 200 nm (Mini-Extruder; Avanti Polar Lipids). Lip-kMA(D12) was prepared by using the cationic dendron-bearing lipid D12 instead of D22. Other materials were mixed at the ratio shown in Table 1 in the same manner as Lip-kMA. An empty liposome control (Lip-con) was prepared similarly, but without the addition of any MA compound. Fluorescent liposomes were prepared by adding a lipophilic dye, 25-[N-[(7-nitro-2-1,3-benzoxadiazol-4-yl)methyl]amino]-27-norcholesterol (NBD-cholesterol) (Avanti Polar Lipids), at 20 μg to the lipids in organic solvents. The endotoxin concentrations in the samples were less than the detection limit (>0.01 EU/ml), as determined using an endotoxin assay kit (ToxinSensor Chromogenic LAL Endotoxin Assay Kit; Genscript, Piscataway, NJ).

### Transmission electron microscopy observation and the measurement of the diameter and zeta potential

The liposomal samples were mixed with a 2% phosphotungstic acid solution, dropped onto a 400-mesh carbon-coated grid and dried in air immediately after the removal of any excess solution. The samples were observed by transmission electron microscopy (TEM) (Tecnai 20; FEI, Hillsboro, OR) at an acceleration voltage of 80kV. Digital images (2048 x 2048 pixels) were taken with a CCD camera (Eagle 2K; FEI) by the Laboratory for Technical Support, Medical Institute of Bioregulation, Kyushu University. The diameter was measured by dynamic light scattering, and the zeta potential was determined by laser-Doppler velocimetry with a ZEN3600 ZETASIZER Nano (Malvern Instruments, Worcestershire, UK).

### Analysis of cellular internalization in MB49 cells

MB49 cells (a murine bladder cancer cell line) were incubated with liposome samples labeled with the green fluorescence marker NBD-Cholesterol. After incubation for the indicated time, the cells were collected and analyzed using a BD FACSVerse flow cytometer (BD Bioscience, San Jose, CA). In the confocal laser scanning microscopy experiments, T24 cells were incubated with liposome labeled with NBD-cholesterol. After a 30-min incubation, the nuclei were stained with 4',6-diamidino-2-phenylindole and the cells were observed by confocal laser scanning microscopy (Leica TCS SP5). Each value represents the mean of triplicate experiments.

### Cytotoxic effects on in vitro culture cells by WST-8 assa*y*

Cells of the murine bladder cancer line MB49 (1×10^3^ cells in 100 μL of culture medium) were seeded in each well of a 96-well plate and pre-cultured overnight. Then, 14.3 μL of PBS or liposomes with or without MAs were added to the culture medium (equivalent to a final MA concentration of 50 μg/ml), and cell viability was assayed with a CCK-8 kit per the kit manufacturer's instructions (Dojindo Molecular Technologies, Mashiki, Japan). The data shown are representative of three independent experiments.

### Antitumor effects on a murine syngeneic graft model

C57BL/6 mice (female, 7–8 weeks old) were subcutaneously inoculated with a mixture of 1×10^5^ MB49 cells and 100 μL of liposome samples (equivalent to 40 μg of MA). Then, 100 μL of reagents was subcutaneously administered on days 3, 5, and 7. The tumor volume was calculated using the following formula: (major axis × minor axis^2^) × 0.52. C3H/HeN mice (female, 7–8 weeks) were subcutaneously inoculated with MBT-2 cells (1×10^5^) by the same protocol as described above. Beige mice and nude mice were subcutaneously inoculated with MB49 cells as described above.

### Histological analysis of MB49 syngraft tumor tissue

For the histopathological analysis, on day 10 the tumors were resected from C57BL/6 mice treated with Lip-con or Lip-kMA. Fixed tissue samples were embedded in paraffin on CT-Pro20 (Genostaff, Tokyo) using G-Nox (Genostaff) as a less-toxic organic solvent for xylene, and sectioned at 4 μm. Tissue sections were de-paraffinized with xylene and rehydrated through an ethanol series and Tris-buffered saline (TBS). Antigen retrieval was performed by microwave treatment with citrate buffer, pH 6.0. Endogenous peroxidase was blocked with 0.3% H_2_O_2_ in methanol for 30 min, followed by incubation with G-Block (Genostaff) and an Avidin/Biotin Blocking Kit (Vector Laboratories, Burlingame, CA).

The sections were incubated with CD4 rat mAb or CD8 rat mAb at 4°C overnight. They were then incubated with anti-Rat IgG Biotin (Vector Laboratories) for 30 min at room temperature, followed by the addition of peroxidase conjugated streptavidin (Nichirei, Tokyo) for 5 min. Peroxidase activity was visualized by diaminobenzidine. The sections were counterstained with Mayer's hematoxylin (Muto, Tokyo), dehydrated, and mounted with Malinol (Muto).

Three independent areas with the most abundant CD4 or CD8 tumor infiltrates were selected separately and digitally imaged. Tumor-infiltrating lymphocytes were counted manually from the digital images displayed on a monitor. All counts were repeated three times by two investigators (M.S. and K.T.) individually, and the mean of the repeat counts was used for the statistical analyses [27].

### Statistical analysis

Comparisons between groups were performed by Dunnett's test or unpaired t-test. The level of significance was set at p<0.05. The statistical analyses were performed using JMP11 software (SAS Institute, Cary, NC).

## Results

### Isolation of MA subclasses of Mycobacterium bovis BCG

**Fig 1:**
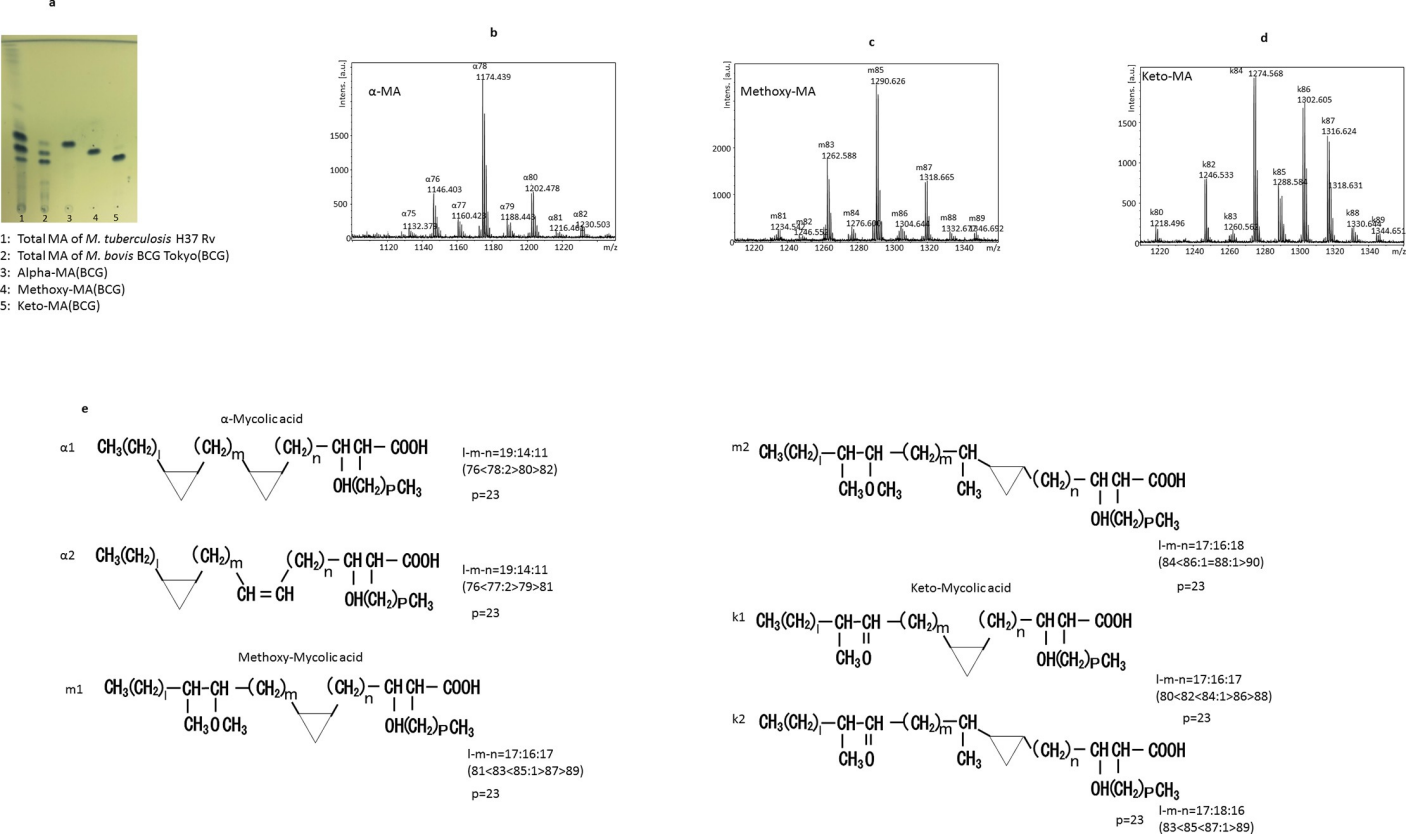
**a: Thin layer chromatography of mycolic acid (MA) subclasses.** The TLC plate was developed with n-hexane-diethyl ether (80:20, v/v; four runs) and visualized by spraying with phosphomolybdic acid. **b–d: MALDI-TOF mass spectra of MA from *M*. *bovis* BCG Tokyo 172, α-MA (b), methoxy-MA (c), and keto-MA (d)**. **e: The chemical structure of MA subclass derived from *M*. *bovis* BCG Tokyo 172**.

The total cellular MA from *Mycobacterium bovis* BCG Tokyo 172 was separated into α-, methoxy-, and keto-MAs by TLC. The relative amount of each MA subclass recovered from the heat-killed bacterial cells was approximately 10:30:60 (w/w/w) (Fig 1A). The MALDI-TOF mass spectra of the total cellular MA showed major clusters of mass ions due to [M+Na]+ (where M is the molecular mass of the MA subclass). We analyzed each molecular species of the MA subclasses separated by silica gel TLC (Fig 1B–1D), and the MALDI-TOF mass spectrometry data of these MA types are summarized in Table 2. The molecular structure of the MA subclass is shown in Fig 1E. Based on the MALDI-TOF mass analysis, the most abundant species of MA was C78 in α-MA, C85 in methoxy-MA, and C84 in keto-MA [28].

**Table 2:** MALDI-TOF mass spectrometric analysis of the cellular MAs from M. *bovis* BCG Tokyo 172.

Subclasses of MA		Total carbon numbers of MAs															
		74	75	76	77	78	79	80	81	82	83	84	85	86	87	88	89
**α**	α1	1117		**1145**		**1173**		**1201**		1229							
	α2		1131		**1159**		**1187**		1215								
**Methoxy**	m1								1233		**1261**		**1289**		**1317**		1345
	m2									1247		1275	1287	1303		1331	
**Keto**	k1							1217		**1245**		**1273**		**1301**		1329	
	k2										1259		**1287**		**1315**		1343

### Development of cationic liposomes incorporating MA

**Fig 2:**
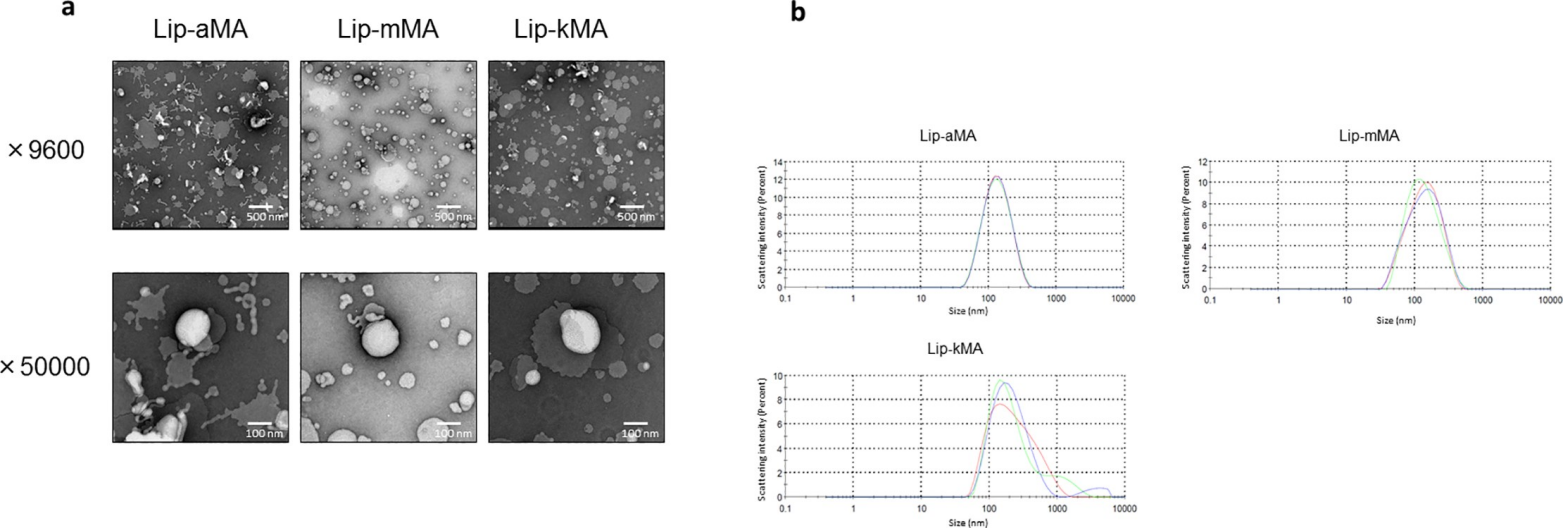
**a, b: Electron micrographs of liposomal MA suspension and the particle distribution.** (a) Lip-aMA was heteromorphic and seemed to be partially disrupted. Lip-mMA and Lip-kMA showed generally round and flat edges. (b) The particle distribution of Lip-aMA, Lip-mMA and Lip-kMA demonstrated by laser-Doppler velocimetry with a ZEN3600.

We first prepared liposome particles by mixing each MA with DOPC and cholesterol. These liposomes had negative surface charges (Table 3A). We used the dendron-bearing lipid D22 to make the liposomes cationic and easily incorporated into cancer cells. The laser-Doppler velocimetry with the ZEN3600 demonstrated that the particle sizes of all types of liposomes were between 120 and 150 nm (Table 3B). We also confirmed the size of the liposome by TEM (Fig 2A). Regarding the surface charges, positive charges were observed in the liposomes containing D22, whereas negative charges were observed in the liposomes without dendron-bearing lipids, indicating that 3% of the dendron-bearing lipids made the liposomes cationic (Table 3A and 3B).

**Table 3:** The diameter, polydispersity index (PdI), and zeta potential of liposomes without a dendrimer (a), and liposomes with D22 (b) measured by a ZETASIZERNano. The values shown are the average of three measurements.

**(a) Without** **dendrimer**	**Dia. (nm)**	**PdI**	**Z potential,** **mV**		
α-	120.5	0.198		−9.17
Methoxy-	118.5	0.222		−7.68
Keto-	154.1	0.134		−6.96
**(b) With D22**				
Lip-con	106	0.144		18.4
Lip-aMA	120.5	0.153		2.96
Lip-mMA	118.5	0.214		8.01
Lip-kMA-	154.1	0.256		10.4

**Fig 3:**
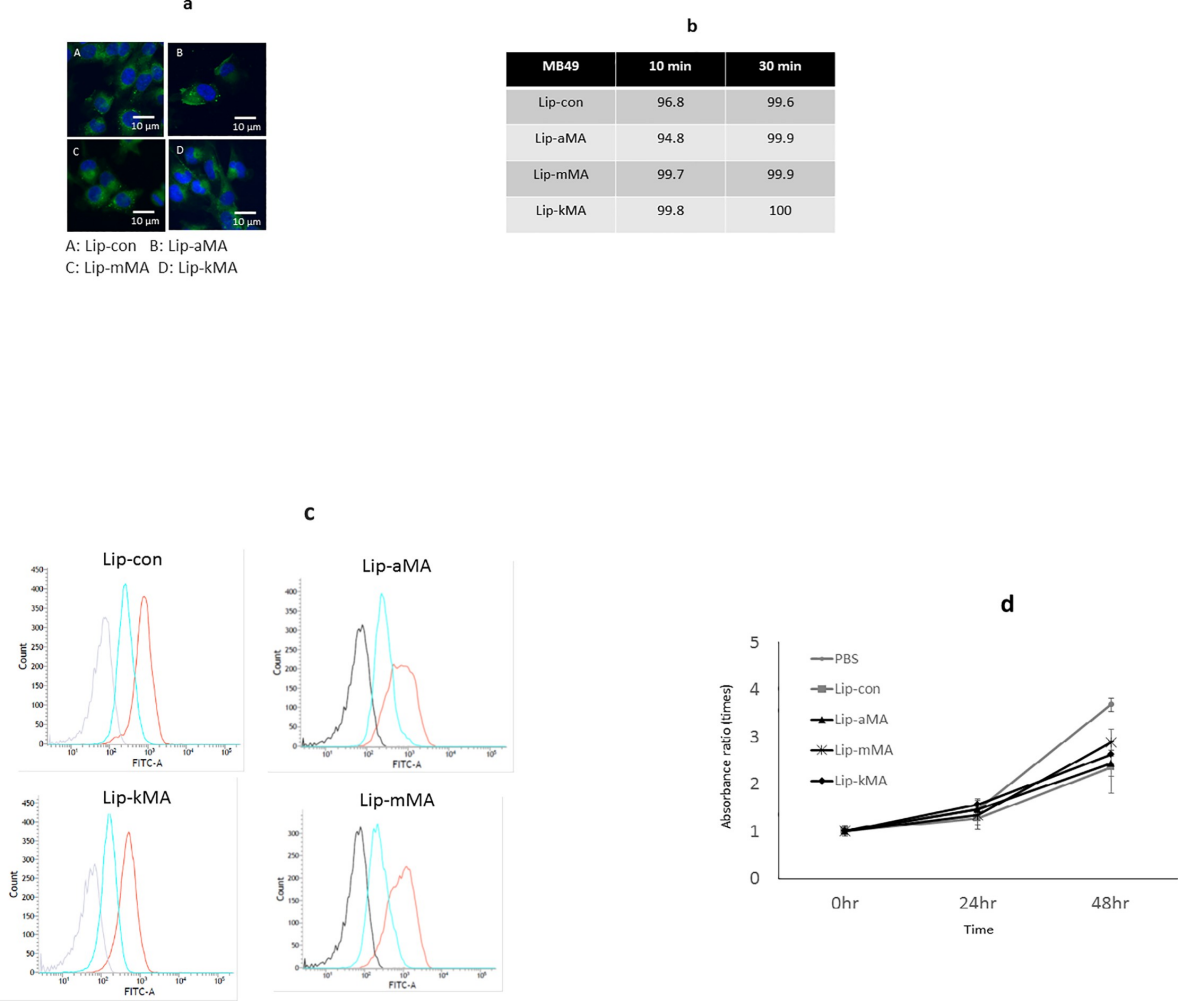
**a, b: Internalization of liposomal MA into bladder cancer cell lines.** Cells of the human bladder cell line T24 and the murine bladder cell line MB49 were incubated with liposome labeled with the green fluorescence marker NBD-cholesterol for the indicated amount of time. Internalization of the liposome was assessed by (**a**) laser confocal microscopy of T24 cells and (**b**) flow cytometry of MB49 cells (%). **c: The cellular association of NBD-labeled liposomes with murine bladder cancer cells analyzed by flow cytometry**. *Black line*: MB49 without liposome. *Blue line*: Liposome without dendrimer. *Red line*: Liposome with dendrimer D22. The experiment was repeated 8 times. **d: The direct cytotoxicity of MA liposome against MB49 cells.** MB49 cells were incubated with liposome containing MA, liposome without MA, or PBS for 48 h in medium without FBS. Cell viability was measured by the WST-8 method. Values represent the absorbance ratio vs. absorbance at 0 h, and were calculated as the mean±SEM of at least three different experiments (P <0.05).

To assess the cellular internalization of these liposomes, we incubated MB49 cells with liposome samples labeled with the green fluorescence marker NBD-cholesterol. Confocal laser scanning microscopy demonstrated strong positive signals in the liposomes with the dendron-bearing lipid D22 (Fig 3A). The flow cytometry analysis also revealed positive signals in >95% of the cells in the liposomes with the dendron-bearing lipid D22, while none of the liposomes without dendron-bearing lipids showed positive signals (Fig 3B and 3C). Finally, we prepared three types of liposome, Lip-aMA, Lip-mMA, and Lip-kMA, by adding each MA to DOPC, cholesterol, and dendron-bearing lipid D22. The Lip-aMA, Lip-mMA, and Lip-kMA contained α-MA, methoxy-MA, and keto-MA, respectively.

### Antitumor activity of MA liposome in the murine syngeneic graft model

Before we assessed the antitumor activity of MA liposomes in the murine syngeneic graft models, we analyzed the direct toxicity of Lip-aMA, Lip-mMA, and Lip-kMA by performing WST assays with a CCK-8 kit. The growth of MB49 cells co-cultured with Lip-aMA, Lip-mMA, or Lip-kMA was not significantly different compared to that of the cells co-cultured with liposomes without MA (Fig 3D).

**Fig 4:**
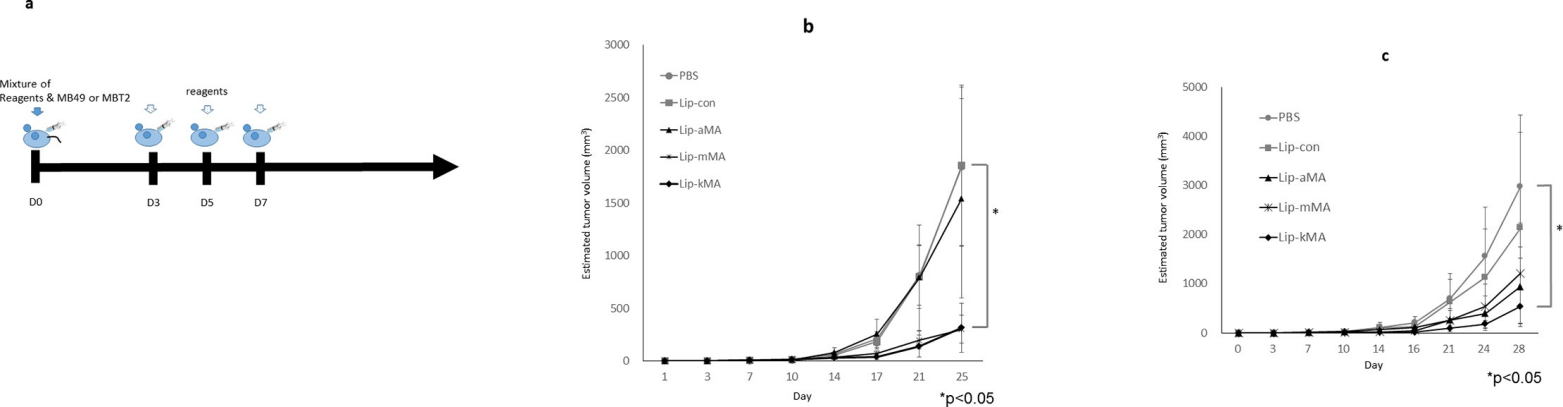
**a: Design of the in vivo study**. Mice were subcutaneously administered a mixture of reagents (PBS, liposome with MA, or liposome without MA) and murine bladder cancer cells (MBT-2 or MB49). Each reagent was then subcutaneously administered on days 3, 5, and 7. Each of these experiments was repeated 5 times and showed similar results. **b: C3H/HeN mice were administered MBT2 cells and treated with each reagent.** At day 25, the growth of the MBT2 tumors was suppressed by treatment with Lip-kMA or Lip-mMA compared to PBS treatment (n = 8–9, p = 0.023, 0.038, respectively). The values are the mean±SEM. *p<0.05, Dunnett's test. **c: The growth of the tumors was recorded**. C57BL/6J mice (n = 5 or 6 for each arm) were administered MB49 cells and treated with each reagent. The growth of the MB49 tumors was suppressed by treatment with Lip-kMA compared to treatment with PBS (day 28, p = 0.028). The activities of Lip-aMA and Lip-mMA were not significant (day 28, p = 0.086, p = 0.16, respectively). The values are the mean±SEM. *p<0.05. Dunnett's test.

We next made a murine syngeneic graft model according to the protocol shown in Fig 4A using two murine bladder cancer cell lines, MBT-2 and MB49. C3H/HeN mice were inoculated with MBT-2 cells, and C57BL/6 mice were inoculated with MB49 cells. The growth of the tumors in the mice was significantly suppressed by Lip-kMA treatment (vs. PBS, p = 0.007), whereas the growth suppression was not significant in the mice treated with Lip-aMA in either model (Fig 4B and 4C). Interestingly, Lip-mMA demonstrated antitumor activity similar to that of Lip-kMA in MBT-2 cells on C3H/HeN mice but weaker activity than Lip-kMA in MB49 cells on C57BL/6 mice.

**Fig 5:**
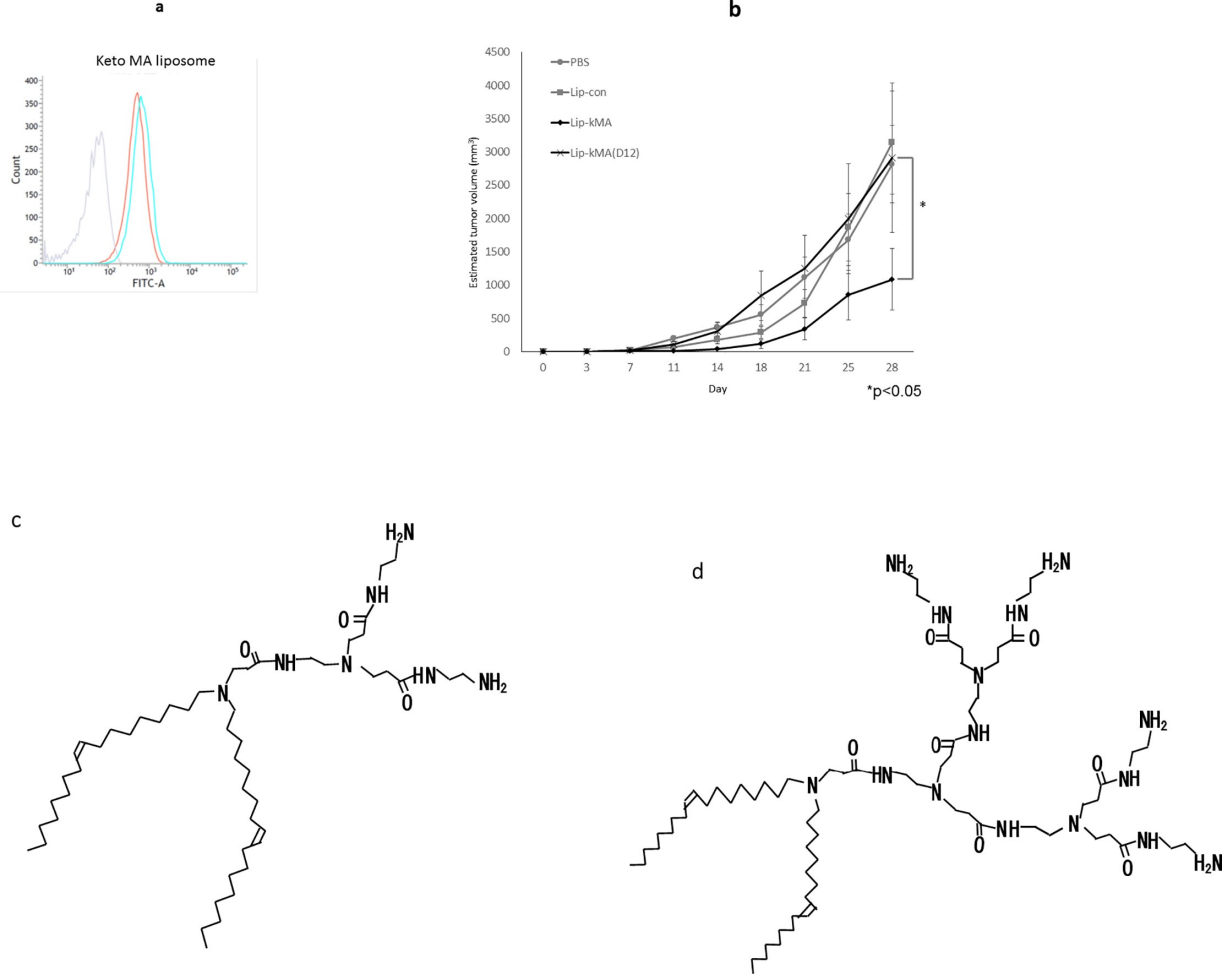
**a: MB49 cells were incubated with liposome labeled with the green fluorescence marker NBD-cholesterol for 10 min.** Internalization of liposome was assessed by flow cytometry. Lip-kMA(D12) was internalized into MB49 cells in a manner similar to Lip-kMA(D22). *Gray line*: Liposome without NBD fluorescence. *Blue line*: Liposome with dendrimer D12. *Red line*: Liposome with dendrimer D22. **b: C57BL/6 mice were administered MB49 cells and treated with each reagent.** At day 28, the growth of the MB49 tumors was significantly suppressed with Lip-kMA compared to Lip-kMA(D12) (n = 5–6, p = 0.04). Values are the mean±SEM. **c: Structure of C18 dialkyl dendrimers.** Dendrimer D12 (C_51_H_101_N_7_O_3_, M.W.860.4). **d: Structure of C18 dialkyl dendrimers.** Dendrimer D22 (C_71_H_141_N_15_O_7_, M.W.1317).

To assess the impact of the types of dendron-bearing lipids on antitumor activity, we prepared Lip-kMA(D12) using another type of cationic dendron-bearing lipid (D12) instead of D22. The particle size and zeta potential of Lip-kMA(D12) were 112 nm and +15.8 mV, respectively, which are comparable to the values of 154.1 nm and 10.4 mV observed for Lip-kMA. Flow cytometry showed that Lip-kMA(D12) was internalized into MB49 cells just as Lip-kMA was (Fig 5A). Lip-kMA(D12), however, showed no antitumor activity in the MB49 murine syngeneic graft model, whereas Lip-kMA exhibited apparent antitumor activity in vivo (Fig 5B).

### T-cell immunity in the antitumor activity of Lip-kMA

**Fig 6:**
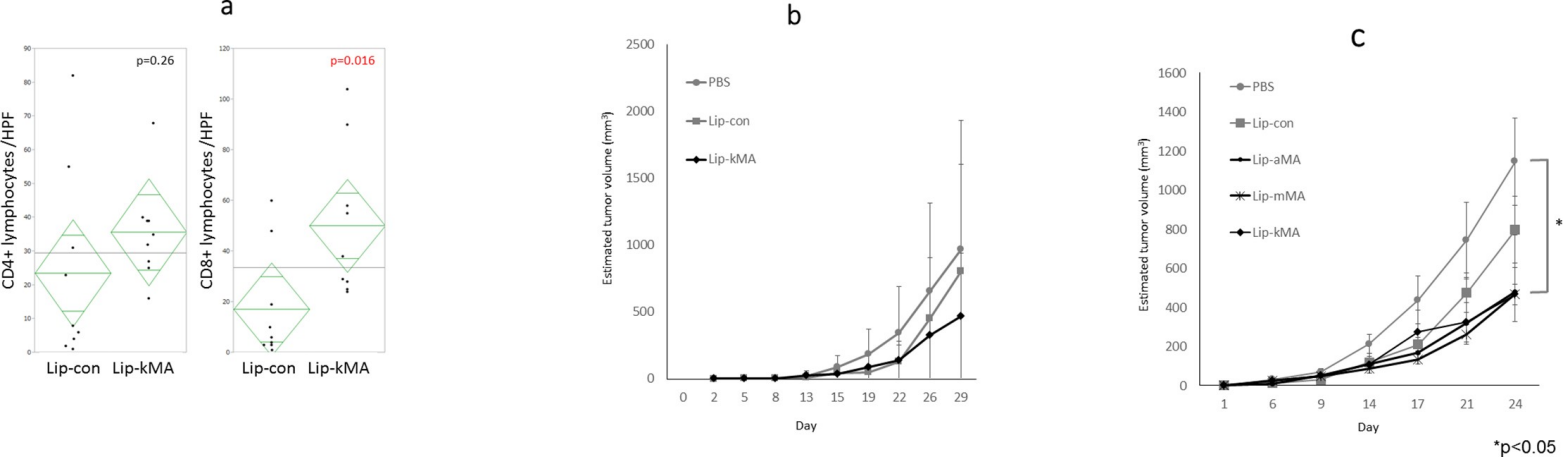
**a: Comparison of CD4/8 infiltrating murine syngeneic tumors.** The number of infiltrating CD8 lymphocytes was significantly higher in the tumors treated with Lip-kMA compared to those treated with Lip-con (50.1 vs. 17.1/HPF, p = 0.016). The difference in the number of infiltrating CD4 lymphocytes was not significant (35.7 vs. 23.6/HPF, p = 0.26). The values are the mean±SEM of at least three different areas (P <0.05). Unpaired t-test. **b: Athymic nude mice (Balb/c nu/nu) were administered MB49 cells and treated with each reagent.** At day 29, the growth of the MB49 tumors was not suppressed with Lip-kMA compared to PBS (n = 5–6, p = 0.65). The values are the mean±SEM. **c: NK cell-deficient beige mice were administered MB49 cells and treated with each reagent.** At day 24, the growth of the MB49 tumors was suppressed by the treatment with Lip-aMA, Lip-mMA, and Lip-kMA compared to that with PBS (n = 5–6, p = 0.023, 0.028, 0.031, respectively). The values are the mean±SEM. *p<0.05. S1 Fig CD4/CD8 immunostaining).

To assess the mechanisms underlying the antitumor activity of Lip-kMA, we histologically analyzed the inoculated tumors of the murine syngeneic graft model. Tumors were resected on day 10 from C57BL/6 mice treated with Lip-kMA or Lip-con. The number of infiltrating CD8 lymphocytes was significantly higher in the tumors treated with Lip-kMA compared to the tumors treated with Lip-con (50.1 vs. 17.1/HPF, p = 0.016). The difference in the number of infiltrating CD4 lymphocytes was not significant (35.7 vs. 23.6/HPF, p = 0.26) (Fig 6A).

For a further assessment of the immune system, we conducted an in vivo experiment with athymic nude mice and beige mice. The antitumor effect of keto-MA liposome essentially disappeared in the nude mice, whereas the effect was partially present in the beige mice with natural killer (NK) activity deficiency (vs. PBS, p = 0.031) (Fig 6B and 6C).

## Discussion

MA is unique to acid-fast bacteria such as *Mycobacterium tuberculosis* and BCG. MA constitutes 40%–60% of the cell envelope by dry weight, and has been shown to play a role in the host immune reaction against *Mycobacterium tuberculosis* [24]. Our present findings demonstrated that cationized liposome containing keto-MA isolated from BCG (Lip-kMA) could induce distinctive tumor cell growth retardation in vivo. To our knowledge, this is the first report to demonstrate that purified MAs have antitumor effects in animal models.

Using single synthetic MA isomers, Vander Beken et al. addressed the relationship between the structure and inflammatory function of this virulence factor. Oxygenated methoxy- and keto-MA with cis-cyclopropane stereochemistry elicited solid to mild inflammatory responses, respectively, whereas α-MA was inert [25]. In the present study, Lip-aMA(D22) showed a lower Z potential (2.96 mV) compared to Lip-mMA (8.01 mV) and Lip-kMA (10.4 mV) (Table 3B). In addition, as shown in Fig 2, the TEM revealed that Lip-kMA and Lip-mMA showed generally round and flat edges, whereas Lip-aMA showed a heteromorphic appearance and seemed to be partially disrupted. These results suggest that the Lip-aMA micelle particle is rather unstable compared to the other two liposomal preparations of MAs, due to the processing of the carboxy terminal to the outer surface of the liposomal particles.

**Fig 7:**
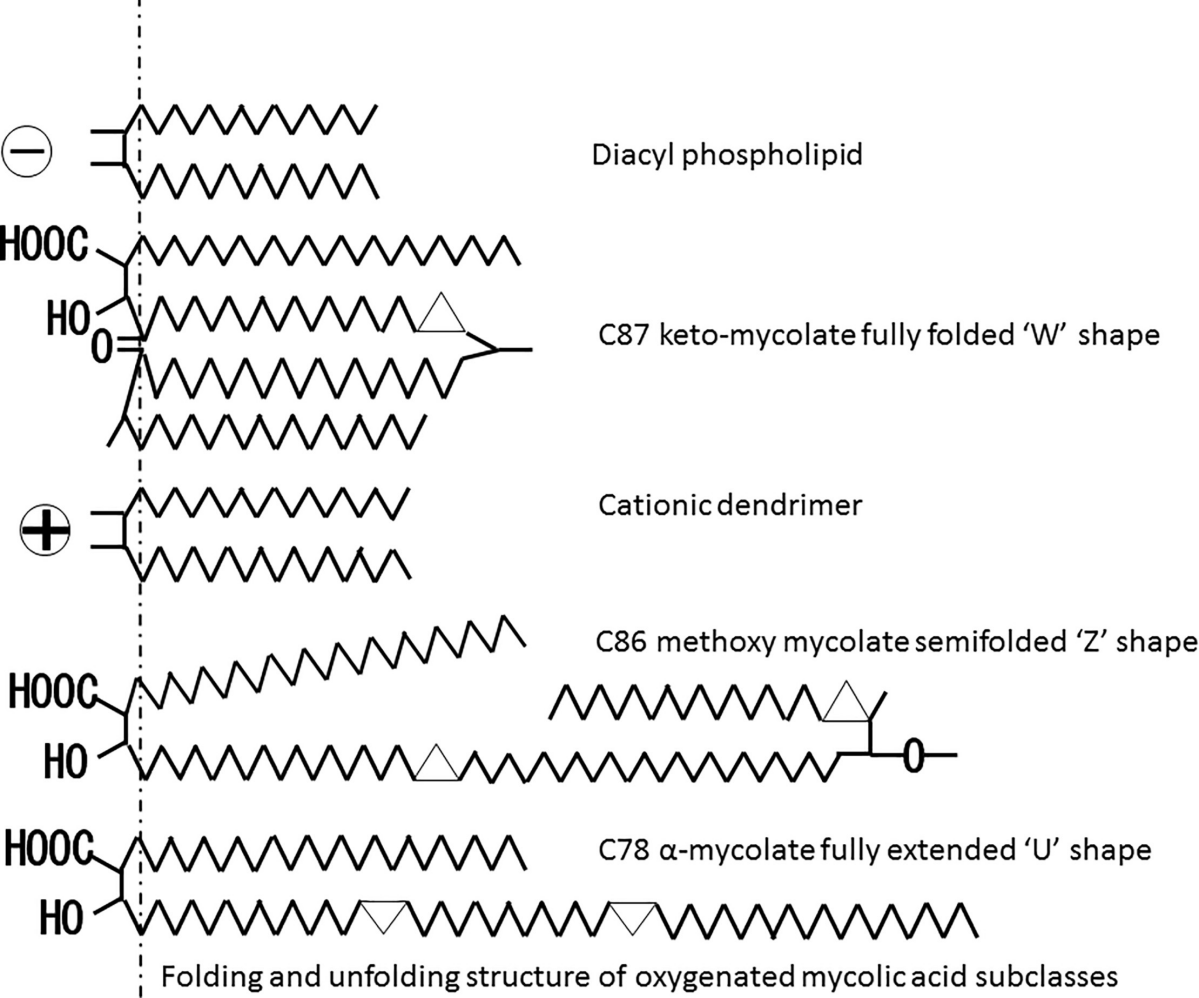
Two-dimensional representative conformations of *M*. *bovis* BCG Tokyo 172 MAs. The C87 keto-mycolate fully folded “W” shape, C86 methoxy mycolate semifolded “Z” shape, and C78 α-mycolate fully extended “U” shape are shown.

We first observed that keto-MA has stronger antitumor activity than α-MA in murine syngeneic graft models. Physiochemical investigations have clearly demonstrated that MAs characteristically adopt distinctly different folded conformations depending on structural characteristics[29–33]. The structural difference between subclasses on the meromycoloyl chain may contribute to the difference in antitumor activity. Since the α-mycolates tend to have an open U-conformation with the two distal chains fully extended [31] [33] (Fig 7), Lip-aMA showed a heteromorphic appearance and seemed to be partially disrupted in the electron micrograph (Fig 2A).

Ketomycolates in *M*. *tuberculosis* predominately fold to yield a compact “W”-conformation, with four chains in parallel [29] [30] [32] [33] (Fig 7). Such tight packing can provide the foundation for an effective hydrophobic permeability barrier in the inner leaflet of the mycobacterial outer membrane. In contrast, methoxymycolates can form W-conformations but also more readily inhabit a range of more extended conformations [29–32] (Fig 7), some of which can be visualized as “U”- or “Z”-shaped [33]. Methoxymycolates show behavior that is intermediate between the α- and keto-mycolates [31] [33]. Although both the W-form of keto-MA and the Z-form of methoxy-MA show antitumor activity, we chose keto-MA because we suspected that the W-form of keto-MA would easily become hydrophilic due to the ease of small liposome formation with this form [34].

Using multiple forms of natural and synthetic MA and MA-specific T cells with different T-cell receptors, Van Rhijin et al. showed that the subclasses differ in their potency for activating MA-specific T-cell clones [23]. Dkhar et al. assessed the transactivation between MA and the host lipid-sensing nuclear receptor testicular receptor 4 (TR4) by using in vitro and in vivo models of granuloma formation, and they stated that a keto-MA-TR4 axis seems to be essential for granuloma formation [35]. In the present study, MAs from BCG were separately incorporated in liposomes. We found that the liposome internalization into MB49 cells did not differ with the MA subclass, but the antitumor activity did differ. Lip-kMA showed antitumor activity in both of the murine syngeneic graft models (C57BL/6J and C3H/HeN), and Lip-aMA showed no activity in either model. Because Vander Beken showed different subsets of inflammatory cells elicited by different subclasses of MA, it is apparent that further investigations are needed concerning the differences in adverse immune responses such as granuloma formation among subclasses, toward the ultimate goal of less-toxic immunotherapy.

As for the mechanisms of the antitumor effects of Lip-kMA, we observed that Lip-kMA could induce antitumor effects in vivo, although it had no impact on cell growth in vitro. In addition, T-cell recruitments were clearly observed 10 days after the administration of Lip-kMA in C57BL/6J mice. These antitumor effects of Lip-kMA were almost completely absent in the athymic nude mice. These results suggest that T lymphocytes play a role in the antitumor activity of Lip-kMA.

T cells that express the CD4 receptors recognize antigens that are present on the surface of host cells by MHC class II molecules. On the other hand, T cells that express the CD8 receptors recognize antigens that are present on the surface of host cells by MHC class I molecules, leading to host cell destruction, and they are therefore also known as cytotoxic T cells. Wang et al. investigated whether both primary CD8 T-cell activation and CD8 T-cell-mediated protection from a *Mycobacterium tuberculosis* challenge could occur in mycobacterial-vaccinated CD4 T-cell-deficient (CD4KO) mice [36]. They found that the CD4KO mice were well protected from *M*. *tuberculosis* at weeks 6 and 12 weeks post-vaccination, and this protection was mediated by CD8 T cells. Their results suggested that both the primary and secondary activation of CD8 T cells is CD4 T-cell-independent and that the maintenance of these CD8 T cells is also independent of CD4 T cells and no longer requires the presence of live mycobacteria. In light of our present findings, we speculate that the antitumor effect may have occurred because CD8 was abundant.

The current state of knowledge regarding innate immune receptors for mycobacteria should also be considered; especially, immunoreceptor tyrosine-based activation motif (ITAM)-coupled C-type lectin receptors (CLRs) [37], and Toll-like receptors (TLRs) [38], which have been shown to recognize mycobacteria-derived cell walls.

Regarding the antitumor effects of BCG, the attachment of BCG to and internalization of BCG into bladder cancer cells triggers the expression of various cytokines and major histocompatibility complex (MHC) class II on the cell surface as a first step in the anti-tumoricity. Various immune cell subsets, including CD4+ and CD8+ lymphocytes, NK cells, granulocytes, macrophages, and dendritic cells, are then recruited around the tumor area and exhibit immunologic reactions against the cancer cells [6]. In a murine model, the depletion of either CD4+ or CD8+ lymphocytes resulted in a loss of BCG-mediated antitumor activity [39]. In a murine bladder cancer model, BCG had no therapeutic effect when administered to athymic nude mice, and its efficacy was restored by an adoptive transfer of BCG-sensitized splenocytes [40]. Our present findings suggest that MA plays a role in the antitumor immunity elicited by BCG therapy.

NK cells are also reported to be one of the essential immune cell subsets in BCG therapy against bladder cancer [41, 42]. Brandau et al. used a syngeneic orthotopic murine bladder cancer model and compared BCG immunotherapy in C57BL/6 wild-type mice, NK-deficient beige mice, and mice treated with anti-NK1.1 monoclonal antibody. BCG therapy was completely ineffective in the NK-deficient beige mice and the mice treated with anti-NK1.1 monoclonal antibody [42]. In the present study, the growth of MB49 tumors in the beige mice was significantly more suppressed by the administration of liposomal MA than by administration of PBS as a control. These results suggest that immune cell subsets other than NK cells may contribute to the activation of T-cell immunity and antitumor activity.

Other immune cell subsets contribute to BCG mechanisms, although we did not examine them in the present study. Neutrophil granulocytes were required for the efficacy of BCG in a murine model [43, 44]. BCG-stimulated macrophages are cytotoxic to bladder cancer cells in vitro [45, 46]. In addition, BCG-exposed dendritic cells can induce T cells to exhibit cytotoxicity against BCG-infected bladder cancer cells in vitro [47, 48]. Cancer cells are also known to play roles such as antigen presentation [49] and cytokine production [11, 50, 51]. In this context, it might be critical to deliver Lip-kMA to immune cells in interstitial tissues as well as to cancer cells.

Regarding the factors of BCG that elicit immune responses, cell wall components of BCG are also thought to be important. The cell wall of BCG contains various components, such as trehalose dimycolate (TDM) [52], cell wall skeleton [16, 20, 53], and lipomannan [54], all of which have been shown to exert antitumor activity. Interestingly, the cell wall skeleton and TDM both contain MA. Lip-kMA may be useful for elucidating the activating mechanism by MA in BCG therapy. Elucidation of the function of MA in this mechanism may lead to a novel BCG-derived immunotherapy.

We compared liposomes using the cationic dendron-bearing lipids D22 and D12, both of which have a polyamide amine dendrimer and lipid double-strand in their molecules. In fact, D22 and D12 differ only in regard to the number of polyamide amine bases [55]. Both Lip-kMA and Lip-kMA(D12) are cationic and were well internalized into tumor cells (Fig 5B). Interestingly, Lip-kMA showed antitumor activity, whereas Lip-kMA(D12) did not. These results suggest that the selection of the cationic dendron-bearing lipid affects the elicitation of an immune response other than internalization into cells. It is difficult to discuss the reason for this difference because there are currently no relevant data. There is thus a need for studies investigating differences in the cationic dendron-bearing lipid, and whether such differences affect the MA processing or antigen presentation in cells. It is noteworthy that not only the voltage of liposomes but also the selection of cationic lipid may alter liposomal immunoactivity.

In the present study, we used a murine bladder cancer syngeneic graft model to evaluate the antitumor activity of MA. Lip-kMA is hydrophilic and can be administered into vessels or the bladder cavity, and we are thus currently conducting an assessment of the antitumor effects of Lip-kMA using a metastatic lung model and an orthotopic bladder tumor model. Our findings will lead to the expansion of therapeutic targets for metastatic disease. At this time, BCG is indicated only for intravesical therapy against bladder cancer, because BCG is a live bacterium and cannot be administered either vessels or orally. A recent study showed that the cell wall component lipomannan induced antitumor immunity against hematologic tumor cells in vivo [54]. Thus the antitumor activity of Lip-kMA against other malignancies should be assessed in the future.

## Conclusions

In conclusion, the immunotherapeutic potential of BCG MA was enhanced by improving its cellular association using a D22 cationic liposome delivery system. D22-encapsulated oxygenated MA, keto-MA, and methoxy-MA showed prominent antitumor activity, but α-MA showed only partial activity. These non-live bacterial agents might serve as more-active and less-toxic alternatives to live BCG or cell wall skeleton in immunotherapy against NMIBC.

## Supporting information

S1 Fig CD4/8 immunostaining. For the histopathological analysis, on day 10 the tumors were resected from C57BL/6 mice treated with Lip-con or Lip-kMA.
(PPTX)

S2 Fig CD4 and CD8 cell count data. Three independent areas with the most abundant CD4 or CD8 tumor infiltrates were selected separately and digitally imaged.
(DOCX)

S3 Fig CD4 and CD8 photograph of immunostaining. (ZIP)

S4 Fig All animal data results. The results of Female C57BL/6, C3H/HeN, nude mice, and Beige mice were included. The result of the types of dendron-bearing lipids on antitumor activity was also included.
(XLSX)
