# Supplementary figures and images for: Cationized liposomal keto-mycolic acids isolated from Mycobacterium bovis bacillus Calmette-Guérin induce antitumor immunity in a syngeneic murine bladder cancer model
Source: PLoS One. 2019 Jan 4;14(1):e0209196. doi: 10.1371/journal.pone.0209196 (PMC6319727; doi:10.1371/journal.pone.0209196)

## Slide 1
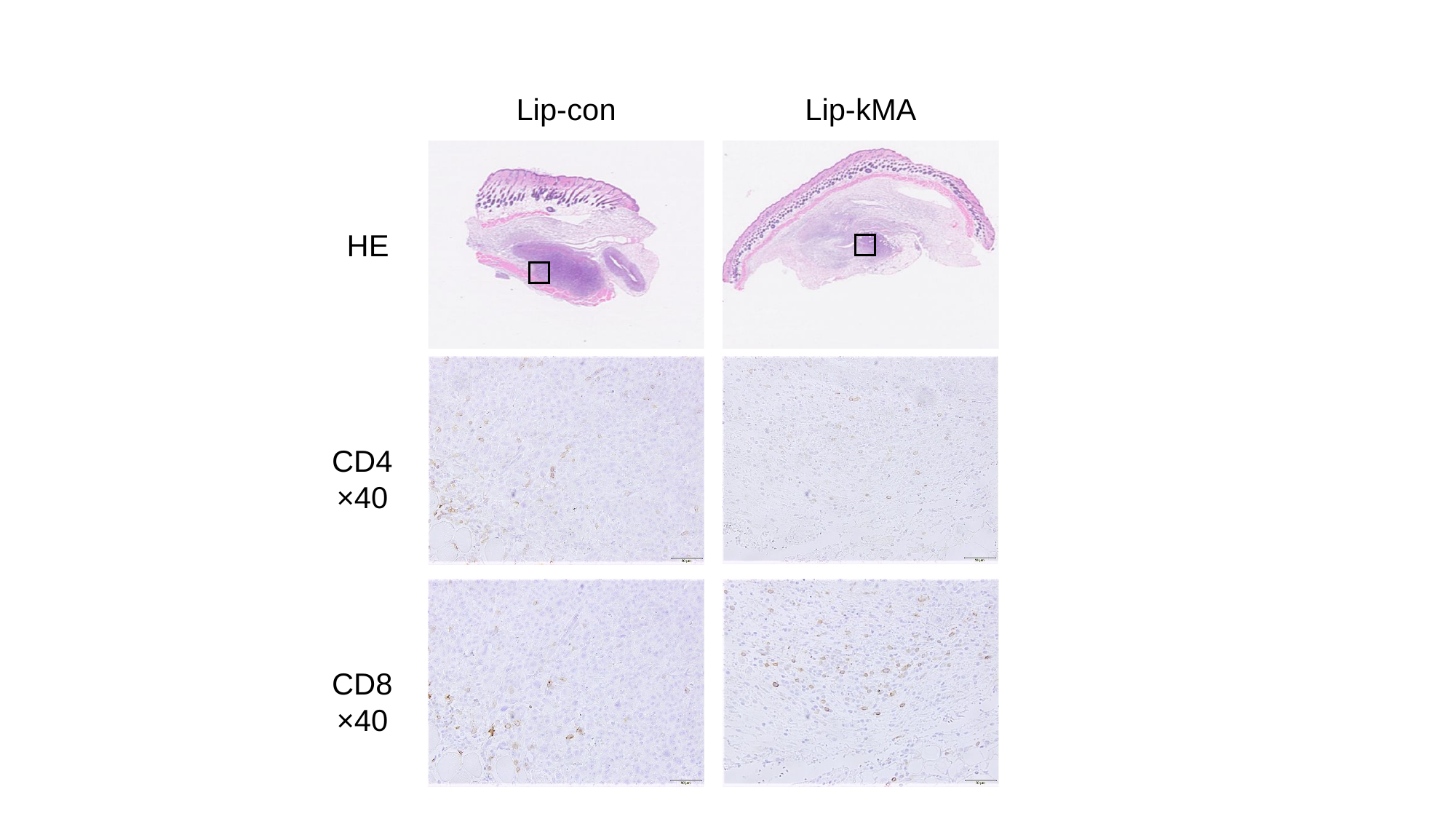

Lip-con
Lip-kMA
HE
CD4
×40
CD8
×40

## Slide 2
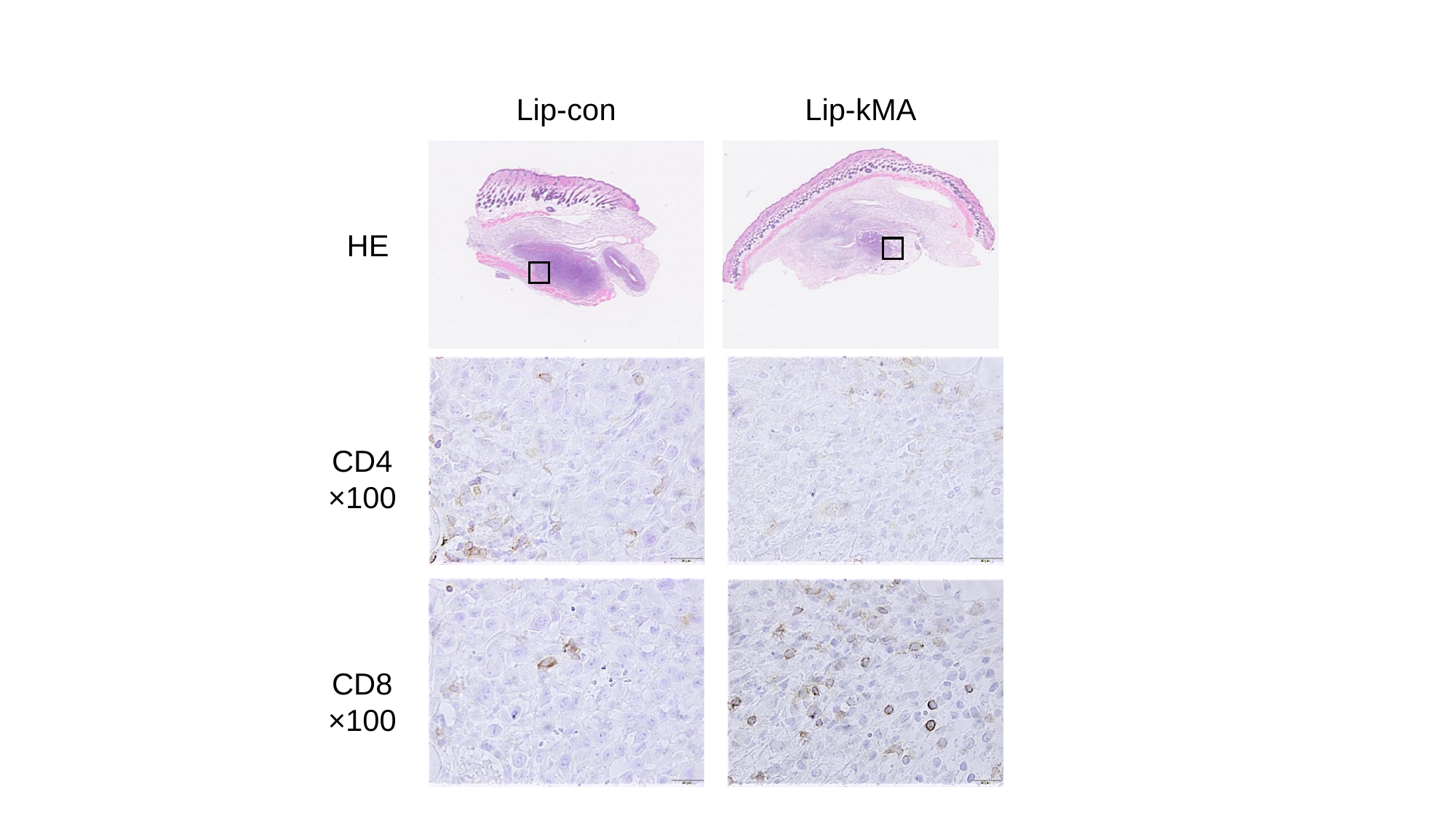

Lip-con
Lip-kMA
HE
CD4
×100
CD8
×100

Supplement: S1 Fig — For the histopathological analysis, on day 10 the tumors were resected from C57BL/6 mice treated with Lip-con or Lip-kMA. (PPTX) [file pone.0209196.s001.pptx]

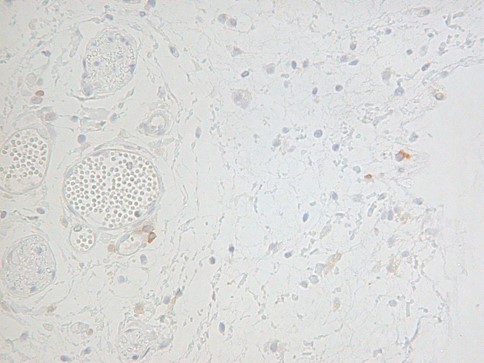

Supplement: S3 Fig — (ZIP) [file pone.0209196.s003.zip › S3 Fig. photograph of immunostaining/c183_CD4_1.jpg]

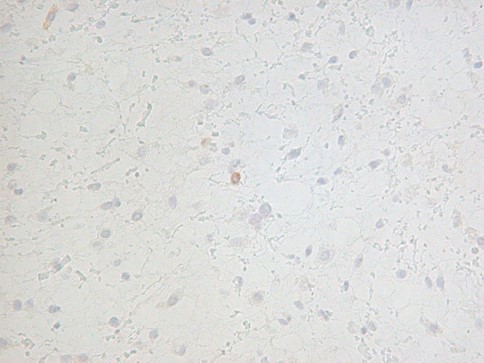

Supplement: S3 Fig — (ZIP) [file pone.0209196.s003.zip › S3 Fig. photograph of immunostaining/c183_CD4_2.jpg]

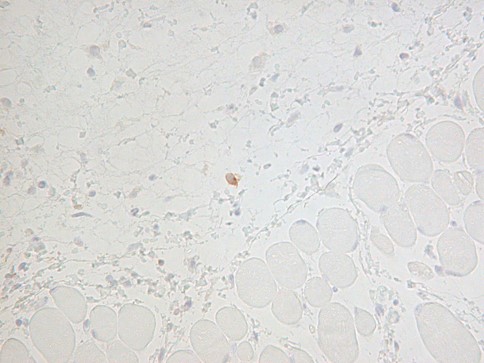

Supplement: S3 Fig — (ZIP) [file pone.0209196.s003.zip › S3 Fig. photograph of immunostaining/c183_CD4_3.jpg]

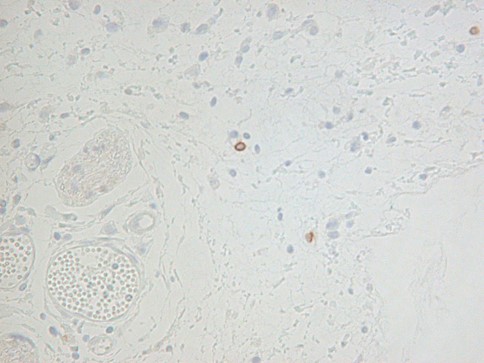

Supplement: S3 Fig — (ZIP) [file pone.0209196.s003.zip › S3 Fig. photograph of immunostaining/c183_CD8_1.jpg]

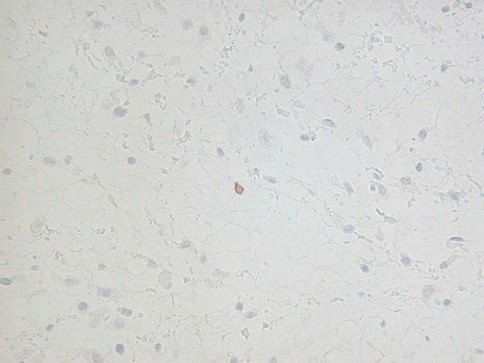

Supplement: S3 Fig — (ZIP) [file pone.0209196.s003.zip › S3 Fig. photograph of immunostaining/c183_CD8_2.jpg]

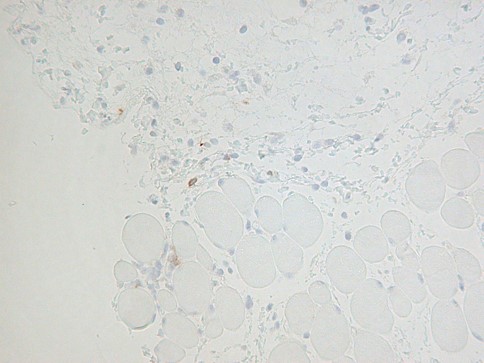

Supplement: S3 Fig — (ZIP) [file pone.0209196.s003.zip › S3 Fig. photograph of immunostaining/c183_CD8_3.jpg]

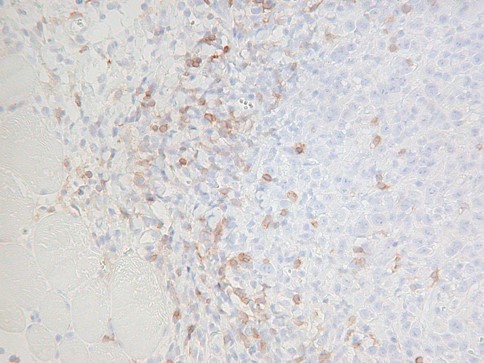

Supplement: S3 Fig — (ZIP) [file pone.0209196.s003.zip › S3 Fig. photograph of immunostaining/c187_CD4_1.jpg]

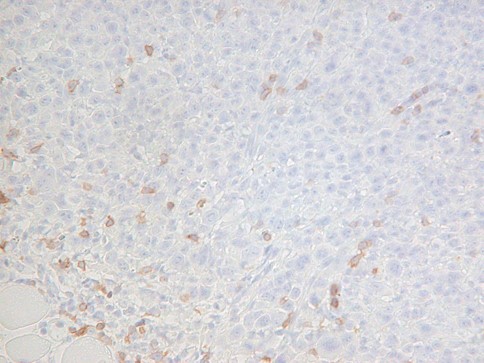

Supplement: S3 Fig — (ZIP) [file pone.0209196.s003.zip › S3 Fig. photograph of immunostaining/c187_CD4_2.jpg]

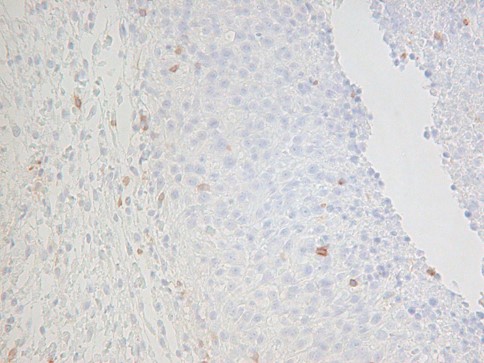

Supplement: S3 Fig — (ZIP) [file pone.0209196.s003.zip › S3 Fig. photograph of immunostaining/c187_CD4_3.jpg]

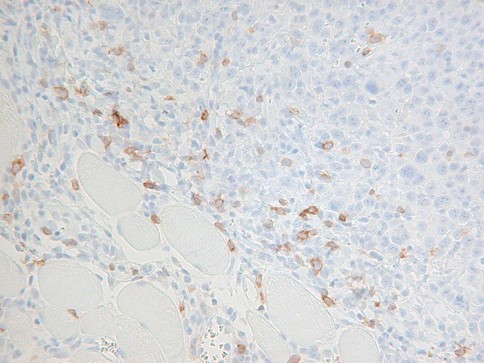

Supplement: S3 Fig — (ZIP) [file pone.0209196.s003.zip › S3 Fig. photograph of immunostaining/c187_CD8_1.jpg]

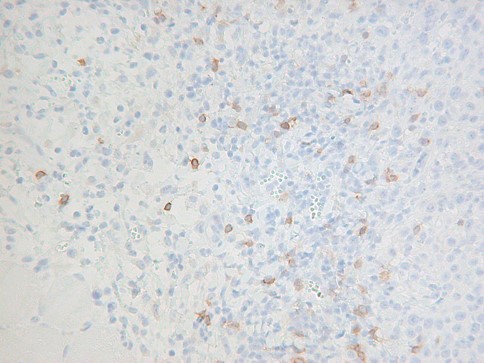

Supplement: S3 Fig — (ZIP) [file pone.0209196.s003.zip › S3 Fig. photograph of immunostaining/c187_CD8_2.jpg]

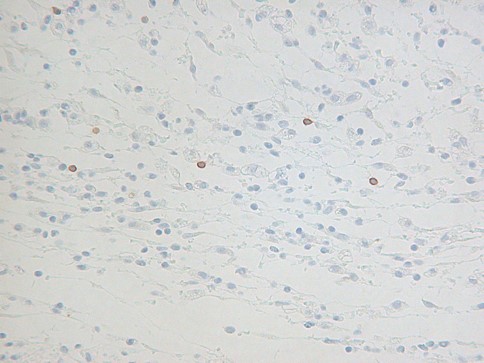

Supplement: S3 Fig — (ZIP) [file pone.0209196.s003.zip › S3 Fig. photograph of immunostaining/c187_CD8_3.jpg]

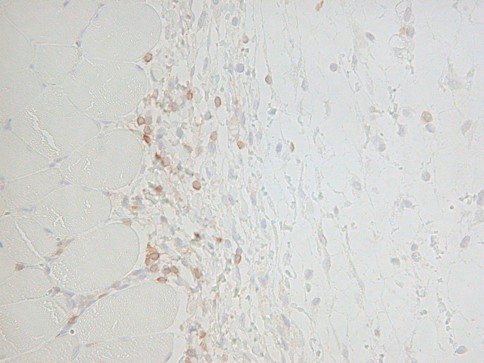

Supplement: S3 Fig — (ZIP) [file pone.0209196.s003.zip › S3 Fig. photograph of immunostaining/c191_CD4_1.jpg]

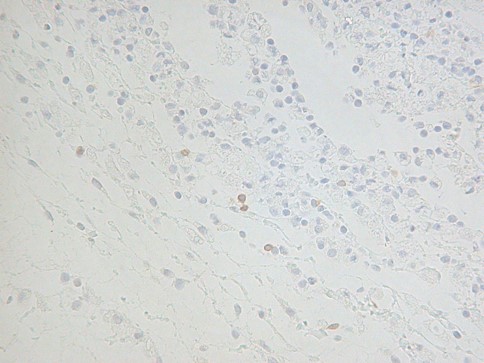

Supplement: S3 Fig — (ZIP) [file pone.0209196.s003.zip › S3 Fig. photograph of immunostaining/c191_CD4_2.jpg]

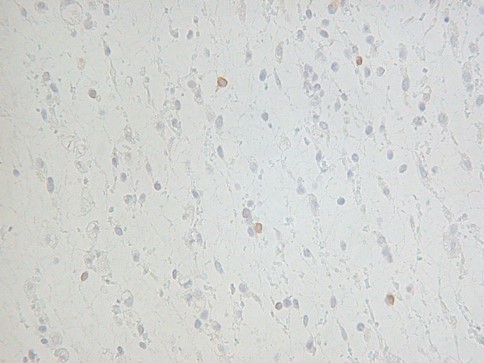

Supplement: S3 Fig — (ZIP) [file pone.0209196.s003.zip › S3 Fig. photograph of immunostaining/c191_CD4_3.jpg]

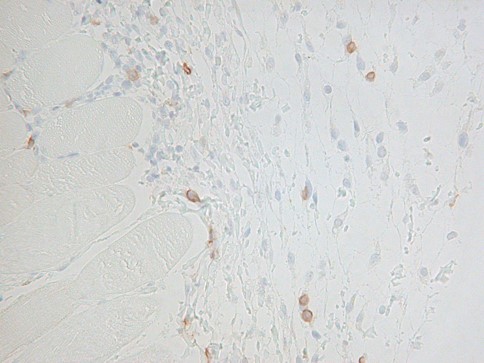

Supplement: S3 Fig — (ZIP) [file pone.0209196.s003.zip › S3 Fig. photograph of immunostaining/c191_CD8_1.jpg]

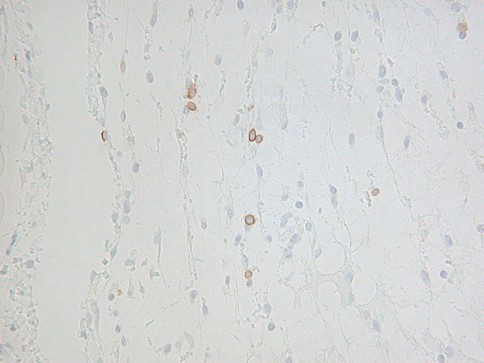

Supplement: S3 Fig — (ZIP) [file pone.0209196.s003.zip › S3 Fig. photograph of immunostaining/c191_CD8_2.jpg]

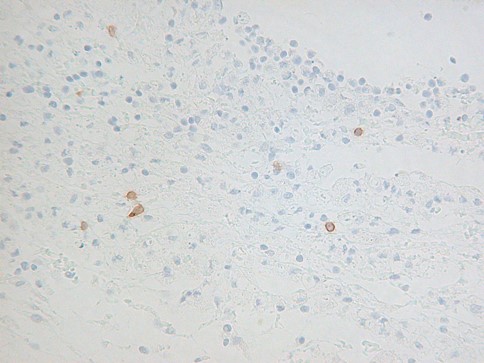

Supplement: S3 Fig — (ZIP) [file pone.0209196.s003.zip › S3 Fig. photograph of immunostaining/c191_CD8_3.jpg]

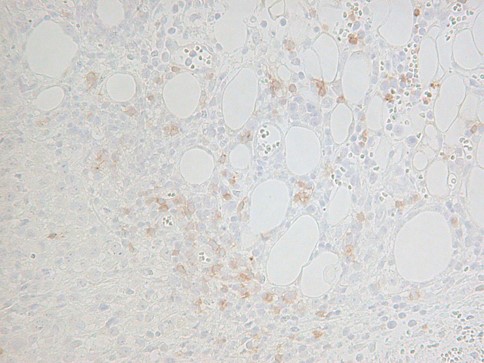

Supplement: S3 Fig — (ZIP) [file pone.0209196.s003.zip › S3 Fig. photograph of immunostaining/k192_CD4_1.jpg]

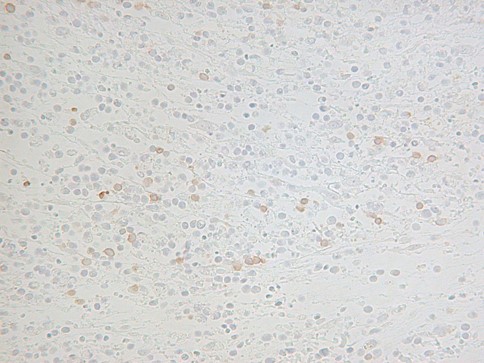

Supplement: S3 Fig — (ZIP) [file pone.0209196.s003.zip › S3 Fig. photograph of immunostaining/k192_CD4_2.jpg]

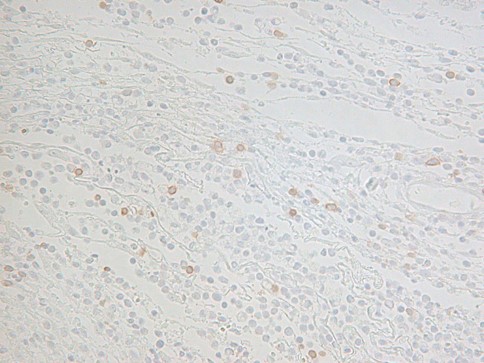

Supplement: S3 Fig — (ZIP) [file pone.0209196.s003.zip › S3 Fig. photograph of immunostaining/k192_CD4_3.jpg]

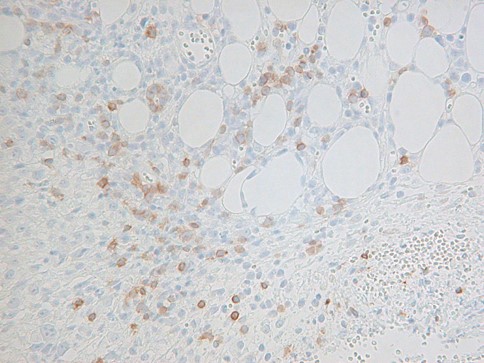

Supplement: S3 Fig — (ZIP) [file pone.0209196.s003.zip › S3 Fig. photograph of immunostaining/k192_CD8_1.jpg]

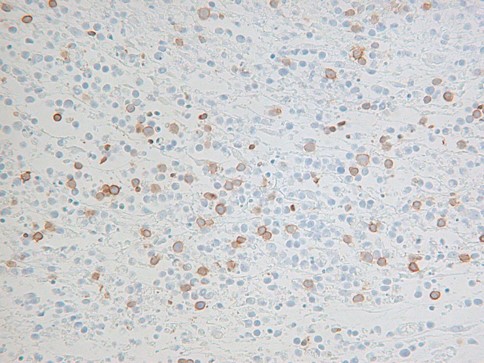

Supplement: S3 Fig — (ZIP) [file pone.0209196.s003.zip › S3 Fig. photograph of immunostaining/k192_CD8_2.jpg]

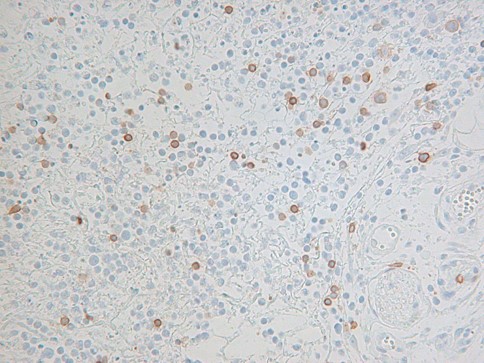

Supplement: S3 Fig — (ZIP) [file pone.0209196.s003.zip › S3 Fig. photograph of immunostaining/k192_CD8_3.jpg]

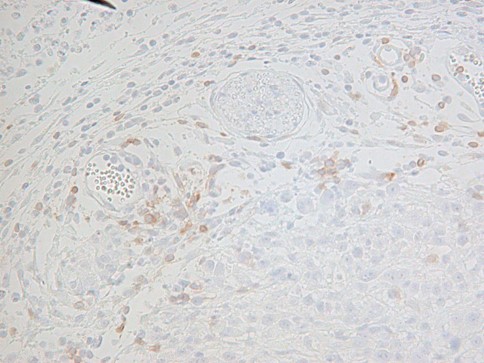

Supplement: S3 Fig — (ZIP) [file pone.0209196.s003.zip › S3 Fig. photograph of immunostaining/k198_CD4_1.jpg]

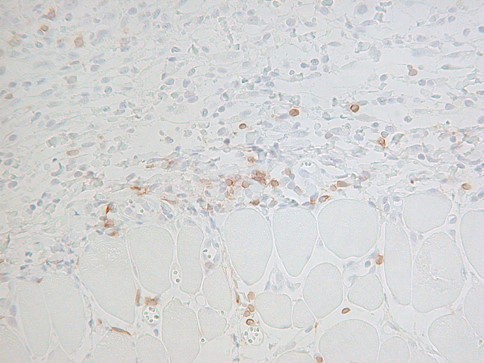

Supplement: S3 Fig — (ZIP) [file pone.0209196.s003.zip › S3 Fig. photograph of immunostaining/k198_CD4_2.jpg]

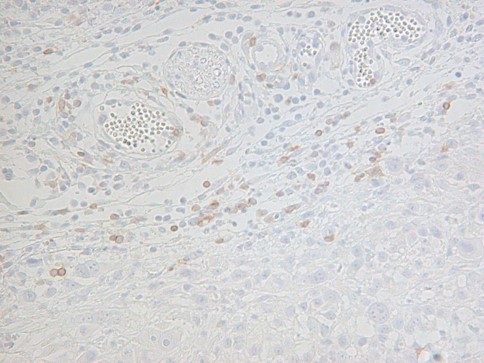

Supplement: S3 Fig — (ZIP) [file pone.0209196.s003.zip › S3 Fig. photograph of immunostaining/k198_CD4_3.jpg]

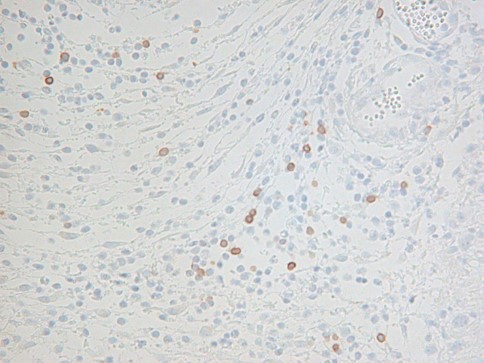

Supplement: S3 Fig — (ZIP) [file pone.0209196.s003.zip › S3 Fig. photograph of immunostaining/k198_CD8_1.jpg]

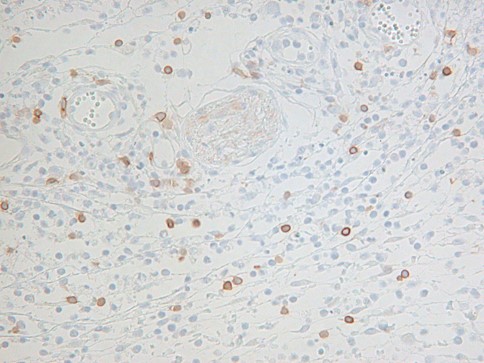

Supplement: S3 Fig — (ZIP) [file pone.0209196.s003.zip › S3 Fig. photograph of immunostaining/k198_CD8_2.jpg]

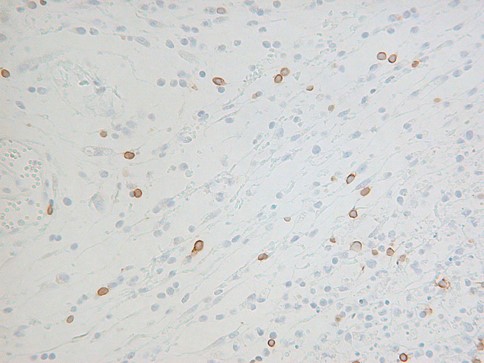

Supplement: S3 Fig — (ZIP) [file pone.0209196.s003.zip › S3 Fig. photograph of immunostaining/k198_CD8_3.jpg]

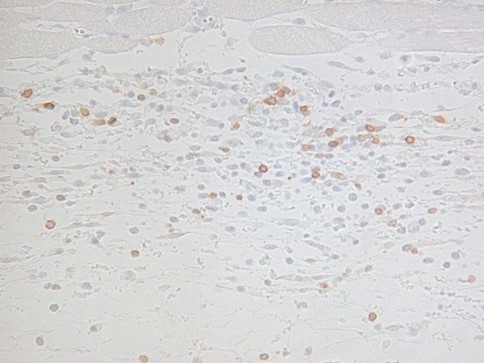

Supplement: S3 Fig — (ZIP) [file pone.0209196.s003.zip › S3 Fig. photograph of immunostaining/k200_CD4_1.jpg]

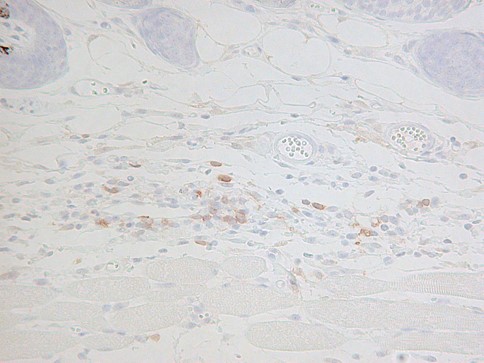

Supplement: S3 Fig — (ZIP) [file pone.0209196.s003.zip › S3 Fig. photograph of immunostaining/k200_CD4_2.jpg]

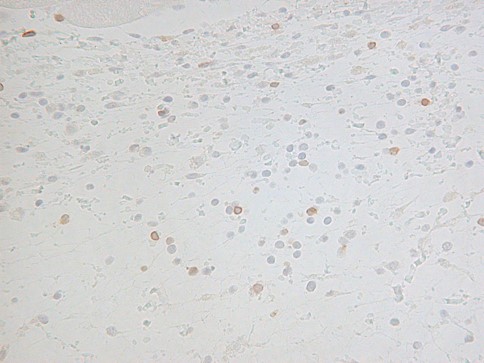

Supplement: S3 Fig — (ZIP) [file pone.0209196.s003.zip › S3 Fig. photograph of immunostaining/k200_CD4_3.jpg]

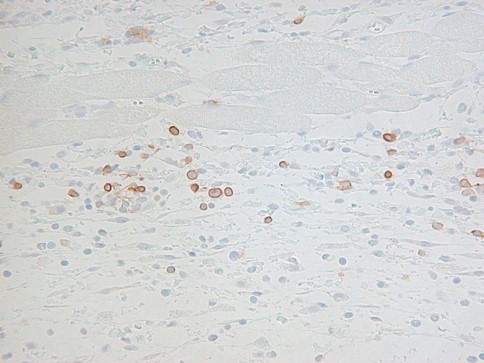

Supplement: S3 Fig — (ZIP) [file pone.0209196.s003.zip › S3 Fig. photograph of immunostaining/k200_CD8_1.jpg]

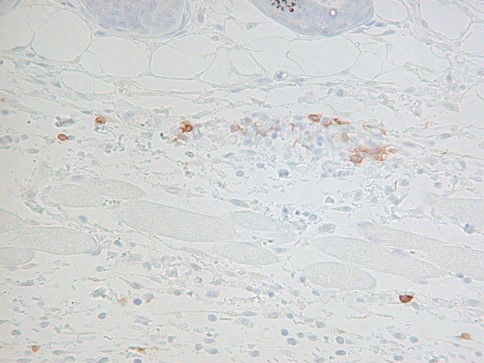

Supplement: S3 Fig — (ZIP) [file pone.0209196.s003.zip › S3 Fig. photograph of immunostaining/k200_CD8_2.jpg]

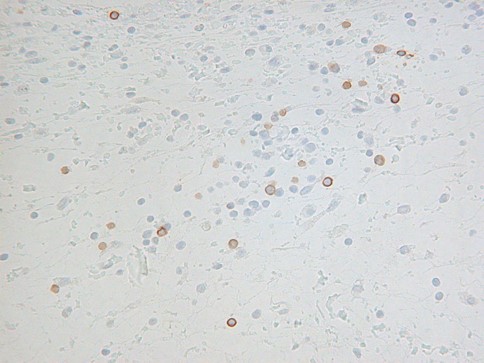

Supplement: S3 Fig — (ZIP) [file pone.0209196.s003.zip › S3 Fig. photograph of immunostaining/k200_CD8_3.jpg]
