# Supplementary material for: Cationized liposomal keto-mycolic acids isolated from Mycobacterium bovis bacillus Calmette-Guérin induce antitumor immunity in a syngeneic murine bladder cancer model
Source: PLoS One. 2019 Jan 4;14(1):e0209196. doi: 10.1371/journal.pone.0209196 (PMC6319727; doi:10.1371/journal.pone.0209196)

CD4

#183: 4+2+1=7 2.33

#187: 82+55+23=160 53.33

#191: 31+8+6=45 15

#192: 68+32+27=127 42.33

#198: 39+35+40=114 38

#200: 39+25+16=80 26.67

Mean：

Control lip 23.55 cells/HPF

Keto lip 35.67 cells/HPF


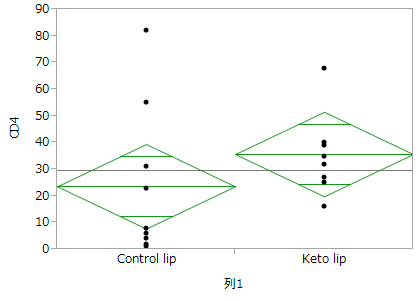


t-statitical analysis


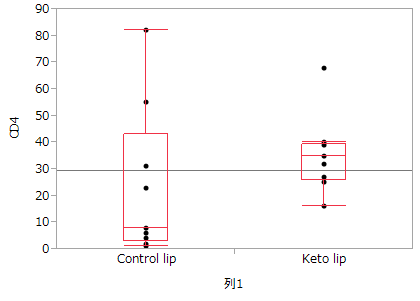


Non-parametric analysis　（Chi-square test）　p=0.0849

CD8

#183: 3+1+3=7 2.33

#187: 60+48+6=114 38

#191: 19+10+4=33 11

#192: 104+90+55=249 83

#198: 28+58+29=115 38.33

#200: 38+25+24=87 29

Mean：

Control lip 17.11 cells/HPF

Keto lip 50.11 cells/HPF


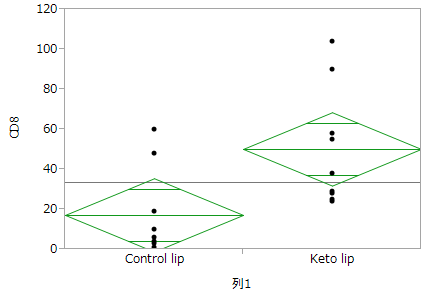


Non-parametric analysis　（Chi-square test）　p=0.0118


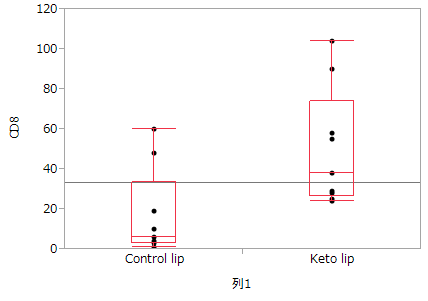

Supplement: S2 Fig — Three independent areas with the most abundant CD4 or CD8 tumor infiltrates were selected separately and digitally imaged. (DOCX) [file pone.0209196.s002.docx]
